# Urine-Based Machine Learning Assay Detects Prostate Cancer

**DOI:** 10.3390/diagnostics16070993

**Published:** 2026-03-26

**Authors:** Marvin S. Hausman, Kyle Ambert, Abhignyan Nagesetti, Francis Buan Hong Lim, Muthukarrupan Swaminathan, Robert F. Cardwell, Obdulio Piloto

**Affiliations:** 1 Genetics Institute of America, Delray Beach, FL 33445, USA; r.cardwell@genlabus.com; 2 PanGIA Biotech, Delray Beach, FL 33445, USAf.lim@pangiabiotech.com (F.B.H.L.);

**Keywords:** non-invasive, diagnostic, urine, liquid biopsy, prostate cancer, random forest

## Abstract

**Background/Objectives**: Prostate cancer testing relies on prostate-specific antigen testing and digital rectal examination, which have limited specificity and face cultural or geographic barriers to access. We developed a non-invasive urine-based liquid biopsy assay using engineered hydrogel arrays and machine learning to detect disease-specific biochemical profiles. **Methods**: We collected voided urine samples from 283 participants at 26 U.S. urology practices prior to prostate biopsy. Random forest classifiers trained on 184 biopsy-confirmed cancer cases and 75 controls analyzed colorimetric signatures. **Results**: Across all Gleason grades (6–10), the assay achieved 97.8% sensitivity and 53.3% specificity. Performance varied by grade: high-grade cancers showed 97.3% specificity, while low-to-intermediate grades demonstrated 94.0% sensitivity. **Conclusions**: This accessible, culturally-appropriate platform could expand prostate cancer detection in diverse populations while reducing unnecessary invasive biopsies.

## 1. Introduction

Prostate cancer remains a leading cause of cancer-related mortality among men globally, yet current screening methods face significant limitations. Prostate-specific antigen (PSA) testing has poor specificity, often leading to unnecessary biopsies in patients with benign prostatic hyperplasia or prostatitis [1,2]. Digital rectal examination (DRE), while complementary to PSA, faces cultural barriers and limited accessibility in many populations [3]. These challenges are particularly acute in low- and middle-income countries, where late-stage detection remains the norm [4]. The need for accurate, non-invasive diagnostic tools has intensified interest in liquid biopsy approaches.

Recent blood-based platforms targeting circulating tumor DNA have shown promise but face sensitivity challenges in early-stage disease [5,6]. Urine offers distinct advantages as a biofluid: it is easily collected, contains metabolites from multiple organ systems, and reflects early physiological alterations in disease states. We developed a urine-based liquid biopsy platform that captures disease-specific biochemical signatures using an engineered hydrogel array analyzed by machine learning—an approach analogous to olfactory sensing, where complex chemical patterns are interpreted computationally rather than targeting predetermined analytes [7,8]. Here, we evaluated the diagnostic performance of this platform, termed the PanGIA Analysis System (PAS), in distinguishing prostate cancer from controls across all Gleason grades. We collected voided urine samples from 283 participants at 26 U.S. urology practices prior to prostate biopsy and analyzed colorimetric signatures using random forest classifiers.

## 2. Materials and Methods

### 2.1. Study Design and Ethics

This prospective validation study evaluated a urine-based liquid biopsy assay for prostate cancer detection. The protocol was approved by Advarra IRB (IORG: 0000635; IRB Registration: 00000971) and followed the methodology described by Lim et al. [9]. All participants provided written informed consent prior to enrollment.

### 2.2. Participant Recruitment and Sample Collection

**Figure 1:**
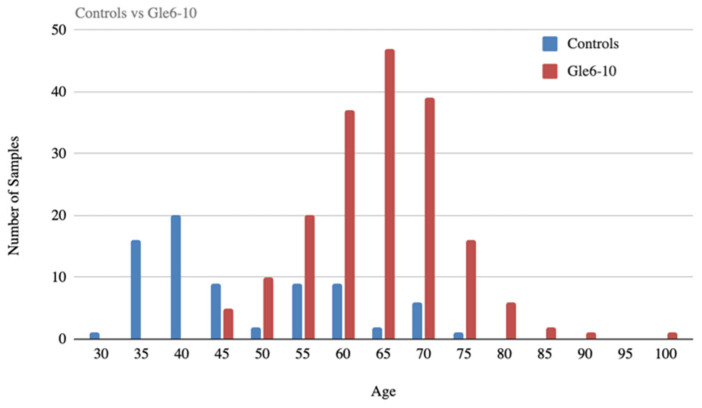
Age distribution of study participants. Number of biopsy-confirmed prostate cancer patients and controls by age group. The prostate cancer cohort (*n* = 184 after outlier removal) had a mean age of 61.2 years (range 30–100 years), with the majority of participants aged 60–70 years (*n* = 123), consistent with known prostate cancer epidemiology.

We recruited 283 participants from 26 urology practices across the United States between August 2020 and November 2023. Inclusion criteria required men scheduled for prostate biopsy based on clinical indication (elevated PSA, abnormal DRE, or suspicious imaging). Exclusion criteria included active urinary tract infection or recent instrumentation. The majority of participants (*n* = 123) were aged 60–70 years (Figure 1). Controls comprised 84 adult males with no clinical indication of prostate disease who provided voided urine specimens. These control donors did not undergo biopsies.

Participants scheduled for needle-guided prostate biopsy received urine collection kits (PanGIA Biotech Inc., Delray Beach, FL, USA) at least one week prior to the procedure. Subjects self-collected first morning-voided urine in pre-labeled containers, then transferred 25 mL into a conical container containing 25 mL of methanol fixative using a disposable pipette. This fixative minimizes microbial growth and minimizes biochemical changes. Specimens were shipped to the central laboratory (Genetics Institute of America, CAP-accredited, CLIA-certified) for processing.

### 2.3. Biopsy Confirmation and Cohort Composition

**Figure 2:**
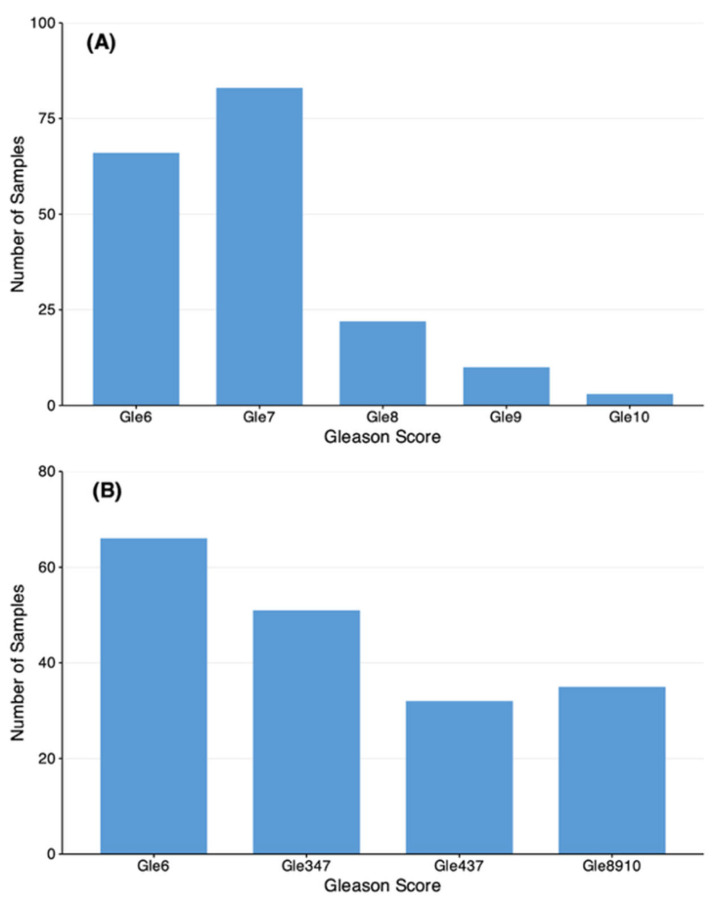
Distribution of prostate cancer cases by Gleason grade and risk stratification. (**A**) Number of cancer-positive participants by Gleason score (*n* = 184). The majority of cases were grades 6 (*n* = 73) and 7 (*n* = 88), with grade 7 subdivided into 3 + 4 = 7 and 4 + 3 = 7 patterns. Higher grades (8–10) comprised the remaining cases. (**B**) Risk stratification of cancer-positive participants. Patients were classified as low-, intermediate-, or high-risk based on Gleason score, PSA levels, and clinical staging criteria.

**Figure 3:**
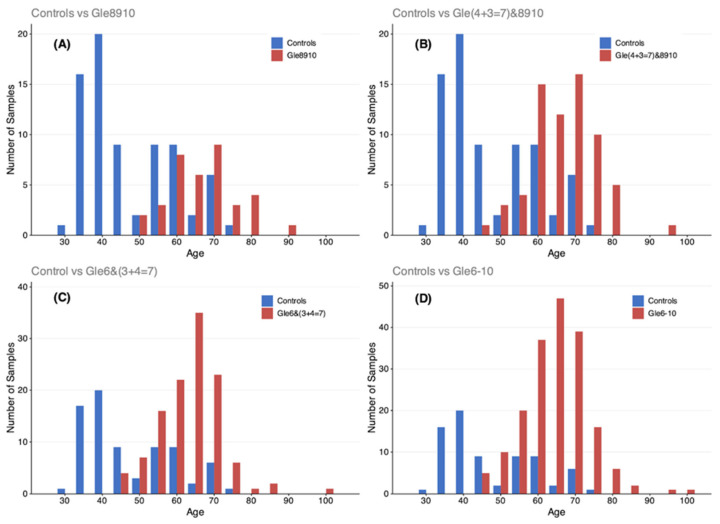
Age distribution of control and cancer-positive cohorts across grade comparisons. Histograms comparing age distributions between control participants (*n* = 75) and cancer-positive subgroups used in the four independent analyses: (**A**) Controls vs. high-grade cancer (Gleason 8–10, *n* = 36), (**B**) Controls vs. intermediate-to-high-grade cancer (Gleason 4 + 3 = 7 & 8–10, *n* = 67), (**C**) Controls vs. low-to-intermediate-grade cancer (Gleason 6 & 3 + 4 = 7, *n* = 117), and (**D**) Controls vs. all grades (Gleason 6–10, *n* = 184). Age matching varied by cancer subgroup, with broader age distributions in analyses including lower-grade cancers.

Prostate biopsy identified 199 cancer-positive participants. Following principal component analysis (PCA)-based outlier removal, 184 cancer cases remained for analysis, distributed by Gleason score as follows: 73 grade 6, 88 grade 7 (subdivided into 3 + 4 = 7 and 4 + 3 = 7), and varying numbers of grades 8–10 (Figure 2A). Cancer-positive participants were stratified by Gleason score, PSA levels, and clinical staging criteria (Figure 2B). Among the controls, 9 were removed as outliers, yielding 75 samples for analysis. Age distributions between the controls and cancer subgroups are shown in Figure 3.

The outliers identified in PCA are due to high variability feature vectors from the feature space as a result of sample processing artifacts. Since PCA looks at the arbitrary rotations resulting from the linear combination of the input dimensions, it is likely that the principal components that account for the high variance are affected by these artifacts. Samples that were inconsistent with others in their cohort were excluded.

### 2.4. NuTec Slide Preparation

NuTec Slides containing 70 engineered hydrogels were manufactured (PanGIA Biotech Inc., FL, USA) as previously described [9]. For each participant, four NuTec Slides were incubated in the urine sample for 10 min, then heated at 250 °C for 5 min to generate colorimetric signatures. The hydrogel array captures metabolites through non-specific binding, producing distinct color patterns based on sample composition.

### 2.5. Image Acquisition and Feature Extraction

Colorimetric signatures were digitized using a conventional flatbed scanner. Images were uploaded to a cloud-based platform (version 2: PanGIA Biotech Inc., FL, USA) for automated feature extraction. Each slide generated a high-dimensional feature vector capturing color intensity, spatial distribution, and pattern characteristics across the 70 hydrogel spots.

### 2.6. Machine Learning Analysis

We employed a supervised learning framework using random forest (RF; sci-kit learn implementation; *n* estimators = 500, all other parameters set to default) classifiers. The dataset was partitioned into training, validation, and test sets using k-fold cross-validation (k = 5). For each fold, the model was trained on the training partition and evaluated on the held-out test partition. This process was repeated five times with different random partitions.

Four independent analyses were conducted: (1) Controls vs. high-grade cancer (Gleason 8–10), (2) Controls vs. intermediate-to-high-grade (Gleason ≥ 4 + 3 = 7 and 8–10), (3) Controls vs. low-to-intermediate-grade (Gleason 6 and 3 + 4 = 7), and (4) Controls vs. all grades (Gleason 6–10).

### 2.7. Performance Metrics

**Figure 4:**
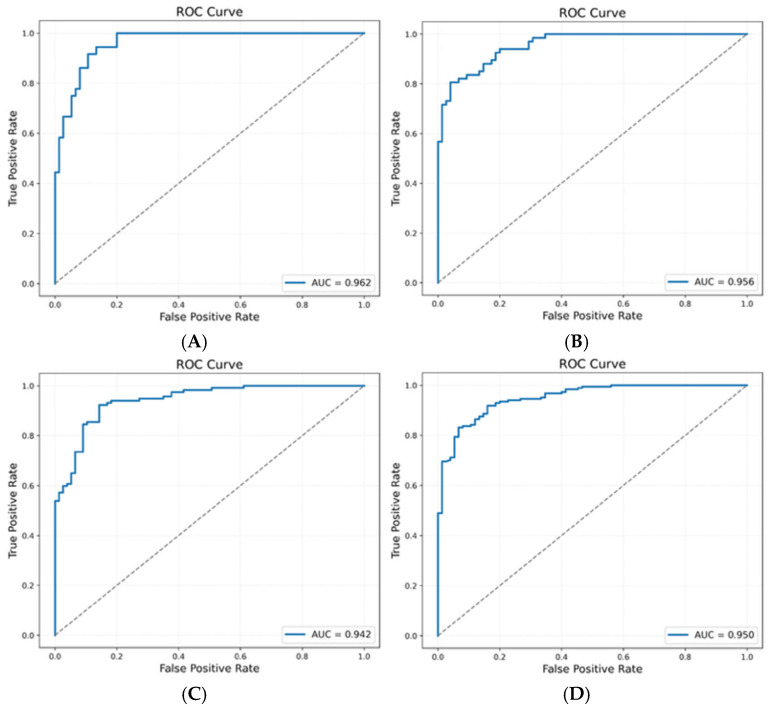
Receiver operating characteristic curves for PAS diagnostic performance across prostate cancer grades. ROC curves showing classifier performance for each grade comparison: (**A**) Controls vs. high-grade cancer (Gleason 8–10, AUC = 0.87), (**B**) Controls vs. intermediate-to-high-grade cancer (Gleason 4 + 3 = 7 and 8–10, AUC = 0.93), (**C**) Controls vs. low-to-intermediate-grade cancer (Gleason 6 and 3 + 4 = 7, AUC = 0.89), and (**D**) Controls vs. all grades (Gleason 6–10, AUC = 0.91). Curves represent mean performance across fivefold cross-validation. Dashed diagonal line indicates the performance of a random classifier (AUC = 0.50).

**Figure 5:**
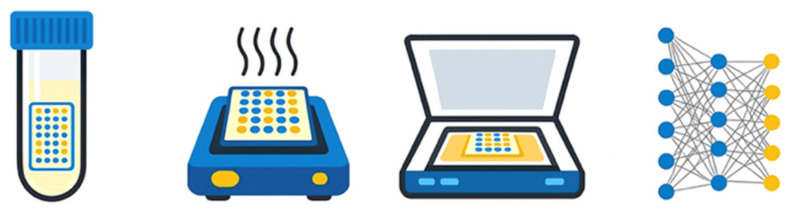
Overview of the PanGIA Analysis System workflow. Schematic depicting the sample processing pipeline: participants self-collected first morning-voided urine, which was fixed with methanol and shipped to the central laboratory. NuTec Slides containing 70 engineered hydrogels were incubated in urine samples for 10 min, then heated at 250 °C for 5 min to generate colorimetric signatures. Slides were digitized using a flatbed scanner, and images were analyzed by a cloud-based machine learning platform using random forest classifiers to distinguish cancer from control samples.

Model performance was assessed using sensitivity, specificity, F1 score, and area under the receiver operating characteristic curve (AUC). Confusion matrices were generated for each analysis. Performance metrics represent mean values across cross-validation iterations. We report aggregated F1 scores to average over any shifts in performance due to underlying data heterogeneity and/or small sample size. ROC curves for all four analyses are shown in Figure 4. An overview of the testing process is summarized in Figure 5, and a summary of all performance metrics for each analysis is summarized in Table 1.

## 3. Results

### 3.1. Diagnostic Performance Across Prostate Cancer Grades

We evaluated PAS performance using a supervised machine learning framework with random forest classifiers. Following quality control and outlier removal, the dataset comprised 259 total samples: 184 biopsy-confirmed prostate cancer cases (Gleason grades 6–10) and 75 controls. Across all cancer grades, PAS achieved 97.8% sensitivity and 53.3% specificity, with an F1 score of 90.2% and an area under the curve (AUC) of 0.91 (Table 1). The classifier correctly identified 180 out of 184 cancer cases, demonstrating robust detection capability across the full disease spectrum.

### 3.2. Grade-Specific Performance

Diagnostic accuracy varied systematically by tumor grade. For high-grade cancers (Gleason 8–10, *n* = 36), the assay achieved 97.3% specificity with 55.6% sensitivity (AUC = 0.87), indicating strong ability to rule out non-cancer samples while maintaining conservative classification thresholds. Intermediate-to-high-grade disease (Gleason 4 + 3 = 7 and 8–10, *n* = 67) showed balanced performance with 92.0% specificity and 79.1% sensitivity (AUC = 0.93). Low-to-intermediate-grade cancers (Gleason 6 and 3 + 4 = 7, *n* = 117) demonstrated the highest sensitivity at 94.0%, though specificity decreased to 68.8% (AUC = 0.89), reflecting greater overlap between molecular profiles of early-stage disease and healthy samples.

Cross-validation across independent data partitions confirmed model reproducibility, with F1 scores consistently exceeding 68% across all grade comparisons. These results indicate that PAS reliably distinguished the prostate cancer-associated biochemical patterns from the controls while showing distinct performance characteristics across the disease continuum.

## 4. Discussion

We developed and validated a urine-based liquid biopsy assay that achieved 97.8% sensitivity for prostate cancer detection across all Gleason grades, with performance characteristics varying strategically by disease stage. High-grade cancers demonstrated 97.3% specificity, minimizing false positives and unnecessary biopsies, while low-to-intermediate-grade disease showed 94.0% sensitivity, enabling reliable early detection. These complementary strengths position PAS as a versatile diagnostic tool adaptable to different clinical contexts—whether prioritizing case detection in screening programs or confirmation in high-risk populations.

Current prostate cancer screening faces significant limitations. PSA testing, while sensitive, has poor specificity (approximately 75%) and positive predictive value (60%), leading to overdiagnosis and unnecessary interventions [9]. Biopsy complications, including bleeding, infection, and sepsis, add substantial healthcare burden [10]. Digital rectal examination remains culturally inappropriate or inaccessible in many populations, particularly limiting screening in low- and middle-income countries where late-stage detection predominates [4]. Recent blood-based liquid biopsy platforms targeting circulating tumor DNA have shown promise but face fundamental challenges: ctDNA is often undetectable during early-stage tumorigenesis, substantially limiting sensitivity when intervention is most effective [11].

Urine-based approaches address these limitations. Unlike blood-based assays, urine reflects early physiological and metabolic alterations, is easily collected without violating cultural norms, and contains diverse analytes from multiple organ systems. Our approach does not target predetermined biomarkers but instead captures complex biochemical signatures [12]—analogous to nanosensor strategies demonstrated for ovarian cancer detection [13]—where machine learning interprets patterns across multiple signals rather than quantifying individual analytes. This design accommodates tumor heterogeneity and inter-patient variability that challenge single-biomarker approaches.

Our cohort demographics (mean age 61.2 years, predominantly Gleason grades 6–7) align with known prostate cancer epidemiology [14,15]. Notably, serum PSA levels in our participants (range 0.4–75 ng/mL) showed no strong correlation with positive diagnosis, consistent with PSA’s well-documented limitations [16]. Emerging evidence suggests that urinary PSA outperforms serum PSA for early detection, with lower urinary concentrations indicating malignancy [17]. Integration of PAS results with serum PSA, urinary PSA, and clinical examination could substantially improve diagnostic precision and risk stratification.

The grade-specific performance patterns we observed have important clinical implications. High specificity for aggressive disease enables confident rule-out of cancer in low-risk patients, while high sensitivity for early-stage disease maximizes detection when curative treatment is most feasible. This adaptability addresses a critical need: current diagnostics struggle to distinguish indolent from aggressive cancers, leading to both over-treatment and missed diagnoses.

Several limitations merit consideration. Our sample size, while sufficient for validation, was modest, and participants were not balanced for age, diet, hydration status, or co-morbidities—factors that may influence urinary metabolite profiles. Future multi-center studies with larger, more diverse cohorts and standardized collection protocols will be essential. Integration of clinical staging data and longitudinal monitoring of treatment response represent important next steps toward clinical implementation, since PAS may be used to associate NuTec Slide profiles with treatment response.

The global burden of prostate cancer demands accessible, culturally-appropriate diagnostic tools. By eliminating requirements for invasive procedures or culturally sensitive examinations, urine-based liquid biopsy could expand screening access in underserved populations while reducing unnecessary biopsies in well-resourced settings. With continued validation and standardization, PAS represents a promising approach to addressing persistent disparities in prostate cancer detection and outcomes.

## 5. Conclusions

With an overall high sensitivity (97.8%), specificity (53.3%) and F1-score (90.2%), this urine based liquid biopsy assay is highly effective at correctly and accurately identifying individuals with prostate cancer without having to resort to invasive testing. Compared to traditional PSA testing, which typically has a sensitivity around 80% and specificity ranging from 20% to 40%, this method excels in providing more reliable results [1,2]. Combined with conventional modalities, the results can be improved to help reduce the risk for overdiagnosis and subsequently the need for invasive biopsies; especially in underserved communities. While this liquid biopsy technology presents a promising diagnostic tool for prostate cancer, with expanded training sets, the technology has the potential to enhance detection, monitoring, and ultimately improve patient outcomes. PAS aims to minimize unnecessary biopsies, diagnose prostate cancer in a culturally sensitive manner, and offer a simple, non-invasive means to detect cancer early when it is most treatable.

## Figures and Tables

**Table 1:** Diagnostic performance of PAS across prostate cancer grade comparisons. Performance metrics for random forest classifiers evaluated using fivefold cross-validation. Four independent analyses compared controls (HC, healthy controls) with cancer-positive subgroups (PCa, prostate cancer) stratified by Gleason score. Metrics represent mean values across cross-validation iterations. AUC, area under the receiver operating characteristic curve.

AUC	F1 Score (%)	Specificity (%)	Sensitivity (%)	Total Samples	Comparison
0.87	68.9	97.3	55.6	111 (75 HC, 36 PCa)	Control vs. Gleason 8–10
0.93	84.1	92	79.1	142 (75 HC, 67 PCa)	Control vs. Gleason 4 + 3 = 7 & 8–10
0.89	87.6	68.8	94	194 (77 HC, 117 PCa)	Control vs. Gleason 6 and 3 + 4 = 7
0.91	90.2	53.3	97.8	259 (75 HC, 184 PCa)	Control vs. All Grades (Gleason 6–10)

## Data Availability

The data presented in this study are available on request from the corresponding author due to privacy concerns.

## Abbreviations

The following abbreviations are used in this manuscript:

PSA	Prostate-specific antigen
DRE	Digital rectal exam
PAS	PanGIA Analysis System
AUC	Area under the curve
HC	Healthy controls
PCa	Prostate cancer
PCA	Principal component analysis
ROC	Receiver operating characteristics
