# Correction: Hausman et al. Urine-Based Machine Learning Assay Detects Prostate Cancer. *Diagnostics* 2026, *16*, 993

**DOI:** 10.3390/diagnostics16172720

**Published:** 2026-08-26

**Authors:** Marvin S. Hausman, Kyle Ambert, Abhignyan Nagesetti, Francis Buan Hong Lim, Muthukarrupan Swaminathan, Robert F. Cardwell, Obdulio Piloto

**Affiliations:** 1 Genetics Institute of America, Delray Beach, FL 33445, USA; r.cardwell@genlabus.com; 2 PanGIA Biotech, Delray Beach, FL 33445, USA; kyle@pangiabiotechnology.com (K.A.); a.nagesetti@pangiabiotech.com (A.N.); f.lim@pangiabiotech.com (F.B.H.L.); muthu@pangiabiotechnology.com (M.S.); o.piloto@pangiabiotech.com (O.P.)

Error in Figure

In the original publication [1], there were mistakes in Figure 4 and its legend. During the editing process, the AUCs were not updated in Figure 4 and its legend. The correct Figure 4 appears below.

Error in Table

In the original publication [1], there were several mistakes in Table 1. During the editing process, the AUCs were not updated in Table 1. The corrected Table 1 appears below.

Text Correction

During the editing process, the AUCs were not updated in the Results section. A correction has been made to Results, 3.1. Diagnostic Performance Across Prostate Cancer Grades, paragraph 1:

We evaluated PAS performance using a supervised machine learning framework with random forest classifiers. Following quality control and outlier removal, the dataset comprised 259 total samples: 184 biopsy-confirmed prostate cancer cases (Gleason grades 6–10) and 75 controls. Across all cancer grades, PAS achieved 97.8% sensitivity and 53.3% specificity, with an F1 score of 90.2% and an area under the curve (AUC) of 0.93 (Table 1). The classifier correctly identified 180 out of 184 cancer cases, demonstrating robust detection capability across the full disease spectrum.

A correction has been made to Results, 3.2. Grade-Specific Performance, paragraph 1:

Diagnostic accuracy varied systematically by tumor grade. For high-grade cancers (Gleason 8–10, *n* = 36), the assay achieved 97.3% specificity with 55.6% sensitivity (AUC = 0.93), indicating strong ability to rule out non-cancer samples while maintaining conservative classification thresholds. Intermediate-to-high-grade diseases (Gleason 4 + 3 = 7 and 8–10, *n* = 67) showed balanced performance with 92.0% specificity and 79.1% sensitivity (AUC = 0.93). Low-to-intermediate-grade cancers (Gleason 6 and 3 + 4 = 7, *n* = 117) demonstrated the highest sensitivity at 94.0%, though specificity decreased to 68.8% (AUC = 0.92), reflecting greater overlap between the molecular profiles of early-stage disease and healthy samples.

The authors state that the scientific conclusions are unaffected. This correction was approved by the Academic Editor. The original publication has also been updated.

## Figures and Tables

**Figure 4:**
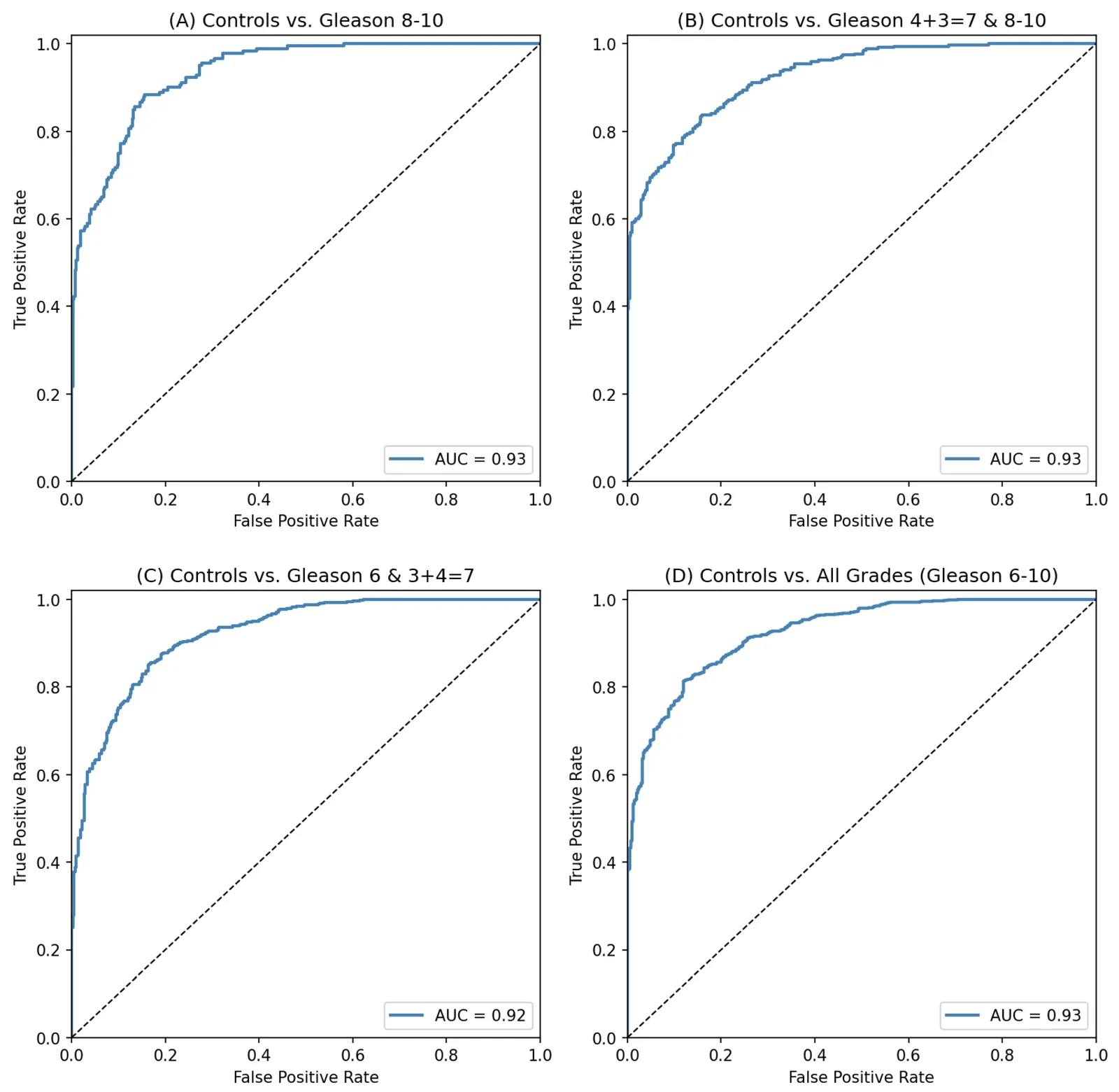
Receiver operating characteristic curves for PAS diagnostic performance across prostate cancer grades. ROC curves showing classifier performance for each grade comparison: (**A**) controls vs. high-grade cancer (Gleason 8–10, AUC = 0.93), (**B**) controls vs. intermediate-to-high-grade cancer (Gleason 4 + 3 = 7 and 8–10, AUC = 0.93), (**C**) controls vs. low-to-intermediate-grade cancer (Gleason 6 and 3 + 4 = 7, AUC = 0.92), and (**D**) controls vs. all grades (Gleason 6–10, AUC = 0.93). Curves represent mean performance across five-fold cross-validation. Dashed diagonal line indicates the performance of a random classifier (AUC = 0.50).

**Table 1:** Diagnostic performance of PAS across prostate cancer grade comparisons. Performance metrics for random forest classifiers evaluated using five-fold cross-validation. Four independent analyses compared controls (HC, healthy controls) with cancer-positive subgroups (PCa, prostate cancer) stratified by Gleason score. Metrics represent mean values across cross-validation iterations. AUC, area under the receiver operating characteristic curve.

AUC	F1 Score (%)	Specificity (%)	Sensitivity (%)	Total Samples	Comparison
0.93	68.9	97.3	55.6	111 (75 HC, 36 PCa)	Control vs. Gleason 8–10
0.93	84.1	92	79.1	142 (75 HC, 67 PCa)	Control vs. Gleason 4 + 3 = 7 & 8–10
0.92	87.6	68.8	94	194 (75 HC, 117 PCa)	Control vs. Gleason 6 and 3 + 4 = 7
0.93	90.2	53.3	97.8	259 (75 HC, 184 PCa)	Control vs. All Grades (Gleason 6–10)
